# AMAZING PAPERS in NEUROSCIENCE: Teaching Neurodevelopment Through the Discovery of Adult Neurogenesis

**DOI:** 10.59390/001c.154518

**Published:** 2025-12-31

**Authors:** Ryan Cunningham

**Affiliations:** 1 Neuroscience University of St Andrews

**Keywords:** Adult Neurogenesis, Neurodevelopment, Hippocampus, Dentate Gyrus, Progenitor Cells, Neuroplasticity, Amazing Papers

## Abstract

Neurogenesis is a critical process in neurodevelopment, contributing to the formation and function of the central nervous system (CNS). Historically, neurogenesis was thought to occur only during embryonic development. However, Eriksson et al. (1998) conducted the first study to confirm neurogenic regions within the adult human brain, using postmortem brain tissue to establish the presence of adult neurogenesis. This study identified adult neurogenesis to be present specifically within the dentate gyrus of the hippocampus, marking a groundbreaking advancement in neurodevelopmental research. The findings from this study highlight the significance of adult neurogenesis, which is now implicated in learning, memory, and in the understanding of neurological disorders such as Alzheimer’s Disease. Integrating this study into undergraduate education introduces students to a pivotal moment in the history of neurodevelopmental research, highlighting its lasting impact on adult neurogenesis research. The study’s manageable length, use of immunofluorescent techniques, and relevance to both basic and clinical neuroscience make it an ideal resource for undergraduate education. Students can develop their skills in critical thinking, scientific literacy, and appreciation of methodological innovation in neuroscience — all while learning and deepening their understanding of the importance of neurodevelopment.

Neurogenesis is a fundamental neurodevelopmental process responsible for generating new neurons - facilitating the formation, development, and functionality of the CNS. During embryonic development, neurons are derived from progenitor cells, which are multipotent stem cells capable of differentiating into various neuronal types in a process known as neurogenesis, as well as glial cells, such as astrocytes and oligodendrocytes, through the process of gliogenesis (Christian et al., 2013; Makrygianni & Chrousos, 2023). The organization and regulation of these progenitor cells are essential to supporting the development and formation of the CNS, guiding its progression to full maturation and enhanced functionality (Hartenstein & Stollewerk, 2015).

Initially, neurogenesis was understood as an essential process occurring only during the early stages of life, during embryonic development (Christian et al., 2013; Ming & Song, 2005). However, the concept of “adult neurogenesis” emerged in the 1960s when studies on rodents revealed neuron generation beyond the embryonic stage, continuing into adulthood. Evidence of neuronal proliferation was first observed in the rat cerebral cortex (Altman, 1962), with subsequent findings in the hippocampus (Altman & Das, 1965). Research with rodents, including rats (Altman & Das, 1965) and rabbits (Guéneau et al., 1982), identified adult neurogenesis as a conserved process within specific neurogenic regions of the hippocampus, notably, the sub-granular zone (SGZ) of the dentate gyrus, which is involved in memory and learning, and the sub-ventricular zone (SVZ) located along the lateral ventricles near the caudate nucleus. In adult rodent studies, progenitor cells have been found to produce new neurons within the hippocampus and olfactory bulb (Kuhn et al., 1996; Ming & Song, 2005), with neurons migrating to the olfactory bulb to replace older neurons (Hussain et al., 2024; Platel et al., 2019). Research in birds has similarly supported adult neurogenesis with evidence of new neurons being produced in the HVC (high vocal center, formerly the hyperstriatum ventrale), a critical area for song learning and memory in avian species (Goldman & Nottebohm, 1983).

Adult neurogenesis was later found to occur in larger mammals, including adult marmoset monkeys (Gould et al., 1998), which paved the way for the first study demonstrating adult neurogenesis in humans. In 1998, researchers examined postmortem human brain tissue from humans who had received BrdU (5-bromo-3′-deoxyuridine), a thymidine analog. BrdU, which can be used to label newly generated cells, was initially administered to track and label tumor growth in cancer patients. However, through postmortem immunohistochemical analyses, this study identified BrdU-labeled cells in the dentate gyrus of the hippocampus. The use of double labeling BrdU with neuronal markers confirmed that newly generated cells within the hippocampus were in fact neurons. This finding confirmed that adult neurogenesis does occur in humans, representing a significant breakthrough in neurodevelopmental research (Eriksson et al., 1998).

The discovery of adult neurogenesis by Eriksson et al. (1998) has profoundly influenced our understanding and study of neurodevelopment in the adult brain. It is now recognized that the hippocampus plays an important role in adult learning and memory processing (Deng et al., 2009; Garthe et al., 2009), further expanding our knowledge of neuroplasticity in adulthood (Saxe et al., 2006). Research has shown that factors like stress and aging negatively impact the proliferation of progenitor cells (Kuhn et al., 1996), while positive influences such as environmental enrichment (Chrusch et al., 2023; Kempermann et al., 1997) and learning (Deng et al., 2009) have been found to enhance adult neurogenesis. Disruption of adult neurogenesis has also been linked to neurological and psychiatric disorders, such as schizophrenia and Alzheimer’s Disease (Ming & Song, 2011; Moreno-Jiménez et al., 2019).

Eriksson et al. (1998) provides academics groundbreaking research that deepens our understanding of neurogenesis, making it an excellent resource for teaching the fundamentals of neurodevelopment to undergraduate neuroscience students. By demonstrating the discovery and existence of adult neurogenesis in humans, Eriksson et al. (1998) serves as an ideal introduction to neurodevelopment which can be used to exemplify how such findings have advanced our understanding of neuroplasticity and developmental processes such as learning and memory, which has further led to implications in neurological and psychiatric disorders.

## RESEARCH SUMMARY

Eriksson et al. (1998) used immunofluorescent labeling on postmortem brain tissue of cancer patients (n = 5) who were treated with BrdU, a thymidine analog used to label newly synthesized DNA during cell replication. BrdU was administered to the patients by intravenous injection, initially for diagnostic purposes to monitor the proliferative activity of tumor cells. To identify newly synthesized neurons, this study also applied neuronal markers NeuN, calbindin (calcium binding protein) and neuron specific enolase (NSE) within neurogenic regions of the brain, such as the dentate gyrus within the hippocampus, as previously identified in mammalian studies (Altman & Das, 1965; Gould et al., 1998; Guéneau et al., 1982). Glial fibrillary acidic protein (GFAP) marker was also used to investigate the presence of astroglia.

Confocal microscopy revealed that most NeuN-positive neurons double labeled with BrdU were located in the granule cell layer (GCL); where neuronal axons extend, and new neurons differentiate (Stanfield & Trice, 1988). These findings represent newly generated neurons within the dentate gyrus of the adult human brain. The same results were shown for those neurons double labeled with BrdU and NSE, across all patients. Cells double labeled for calbindin and BrdU were present in the adult SGZ, GCL and hilus within the hippocampal dentate gyrus, while GFAP-positive, BrdU-labeled cells indicated the presence of glial cells. BrdU-positive cells were also observed within the SVZ, though they did not express GFAP or NeuN. This finding supports the idea that the human brain contains progenitor cells, which migrate from the SVZ before differentiation (Kuhn et al., 1996). This finding aligns with later studies confirming progenitor cell migration from the SVZ to the olfactory bulb, where they compete for integration and replace older neurons (Platel et al., 2019).

Finally, confocal z-stacking analyses and brightfield staining both confirmed that the BrdU-positive neurons in the hippocampus showed a morphology similar to adult rodent neurons, strongly supporting the study’s conclusion that neurogenic regions exist in the adult human brain and that neurogenesis occurs in adults.

## TEACHING VALUE AND GUIDELINES

Eriksson et al. (1998) provides an introduction for undergraduate students to understand neurogenesis and its importance in neurodevelopment. As the first study to confirm adult neurogenesis in humans, it serves as an excellent starting point for a class on neurodevelopment.

Presenting this paper at the beginning of a neurodevelopment-focused course would allow students to grasp the fundamental and historical context of adult neurogenesis early on. Ideally, the paper could be assigned as essential reading for a lecture on neurogenesis. The lecture might begin with an overview of embryonic neurogenesis, then transition to animal studies that initially suggested the presence of adult neurogenesis. Toward the end of the lecture, Eriksson et al. (1998) could be discussed in class, focusing on the study’s methodology and its pivotal findings - particularly the discovery of adult neurogenesis in humans. This discussion would serve as a teaching point, to illustrate how theories of adult neurogenesis developed through animal studies were validated in human research. Students could examine the paper’s strengths and limitations, such as its small sample size, timing of BrdU labeling, postmortem tissue, and study rationale, making this an ideal exercise for developing critical analysis skills. Given that the paper is relatively short (only five pages), it provides a manageable starting point for students to practice and learn critical reading skills in scientific research, before moving on to more complex studies later in the module. The use of immunofluorescent labeling by Eriksson et al. (1998) will also give students the opportunity to analyze fluorescent images, which can be a teaching point for understanding this protocol, the use of double-labeling and the use of cellular markers to label neurogenesis.

To consolidate the teaching of adult neurogenesis, a supplementary YouTube video (Nymus 3D, 2017) could be presented, highlighting principles of neurogenesis alongside examples of sensory processing and potential applications of adult neurogenesis to neurological conditions, such as depression and anxiety. This video would help students appreciate the importance of adult neurogenesis and emphasize the long-standing impact of Eriksson et al. (1998)’s findings. Given that this video (Nymus 3D, 2017) discusses the applications of adult neurogenesis, this can serve as a starting point for the students to investigate how adult neurogenesis has influenced our understanding of the adult brain. From here, students can work in small groups, where they can present and discuss a research paper of their choice (although, related to adult neurogenesis) to their peers in a journal club format. Possible topics include the hippocampus’s role in learning and memory during adulthood (e.g., Deng et al., 2010) or the disruption of neurogenesis in conditions like Alzheimer’s Disease (e.g., Moreno-Jiménez et al., 2019). This activity encourages students to explore the literature more in-depth, while developing skills in scientific communication and presentation.

The length of this study can also serve as a valuable teaching point for students, demonstrating that scientific research does not need to involve numerous experiments or a large sample size to create groundbreaking research. While Eriksson et al. (1998) included only five participants, which may seem like a quantitative limitation, the study nonetheless produced groundbreaking findings, provided important answers to established theories in neurogenesis and greatly advanced our understanding of neurodevelopment.

## AUDIENCE

This paper is ideally suited for teaching more advanced undergraduate students at the beginning of a module or course on neurodevelopment. Eriksson et al. (1998)’s findings lay foundational knowledge of neurogenesis and are generally easy to understand; however, to fully engage with and understand the study, students should have a basic proficiency in understanding immunofluorescent techniques and imaging. This background knowledge will enhance their comprehension of the study’s use of markers and labeling techniques for distinguishing cell types (e.g., NeuN as a neuronal marker, GFAP as a glial marker).

Alternatively, this could be introduced to beginner to intermediate level undergraduate classes, provided students have a strong grasp of immunofluorescent methods, as its historically significant findings in neuroscience may spark interest in neurodevelopment as students begin to narrow their research focus, before progressing to more advanced levels of study.

As previously mentioned, this study also serves as an excellent resource and opportunity to develop key skills in neuroscience, such as critical reading and evaluation of research strengths and limitations. As such, it could be used in class to foster these skills early on, supporting students as they advanced in their academic career.

## CONCLUSION

Eriksson et al. (1998) provided the first study to confirm adult neurogenesis in humans. This was previously thought to only occur during embryonic development, but this study provides evidence for neurogenesis to persist throughout adulthood in humans. This study can be used to teach undergraduate neuroscience students about the history of neurogenesis research, and offers a foundation for understanding neurogenesis in adults. Additionally, it provides a basis for exploring the implications of adult neurogenesis in research such as learning, memory, and neurological disorders. Overall, this study by Eriksson *et al.* (1998) is a pioneering contribution that remains fundamental to both research and education in neurodevelopment.
